# Consensus paper on *Candida auris* by Türkiye EKMUD, ID-IRI, THSK of Ministry of Health of the Republic of Türkiye, KLIMUD, TMC, TARD, and TYBD

**DOI:** 10.55730/1300-0144.6059

**Published:** 2025-02-02

**Authors:** Canan AĞALAR, Hakan ERDEM, Yasemin ÇAĞ, Bilgin ARDA, Recep BALIK, Aliye BAŞTUĞ, Burcu DALYAN CİLO, Nurettin ERBEN, Esma ERYILMAZ EREN, Gülşen İSKENDER, Ayşe KALKANCI, Nilgün KARABIÇAK, Zeliha KOÇAK TUFAN, Emine PARLAK, Nagehan DİDEM SARI, Murat SAYIN, Seniha ŞENBAYRAK, Recep TEKİN, Ayşegül ULU KILIÇ, Tuğhan UTKU, Ömrüm UZUN, Göknur YAPAR TOROS, Sevtap ARIKAN AKDAĞLI

**Affiliations:** 1 Department of Infectious Diseases and Clinical Microbiology, Faculty of Medicine, Fenerbahçe University, İstanbul, Turkiye; 2 Department of Infectious Diseases and Clinical Microbiology, Faculty of Medicine, Health Science University, Gülhane Training and Research Hospital, Ankara, Turkiye; 3 Department of Infectious Diseases and Clinical Microbiology, Faculty of Medicine, İstanbul Medeniyet University, İstanbul, Turkiye; 4 Department of Infectious Diseases and Clinical Microbiology, Faculty of Medicine, Ege University, İzmir, Turkiye; 5 Department of Infectious Diseases and Clinical Microbiology, Haydarpaşa Numune Training and Research Hospital, İstanbul, Turkiye; 6 Department of Infectious Diseases and Clinical Microbiology, Faculty of Medicine, Health Science University, Ankara Bilkent City Hospital, Ankara, Turkiye; 7 Department of Medical Mycology, Faculty of Medicine, Health Science University, Bursa High Specialty Training and Research Hospital, Bursa, Turkiye; 8 Department of Infectious Diseases and Clinical Microbiology, Faculty of Medicine, Eskişehir Osmangazi University, Eskişehir, Turkiye; 9 Department of Infectious Diseases and Clinical Microbiology, Faculty of Medicine, Health Science University, Kayseri City Training and Research Hospital, Kayseri, Turkiye; 10 Department of Infectious Diseases and Clinical Microbiology, Faculty of Medicine, Health Science University, Dr. Abdurrahman Yurtaslan Ankara Oncology Training and Research Hospital, Ankara, Turkiye; 11 Department of Medical Microbiology, Faculty of Medicine, Gazi University, Ankara, Turkiye; 12 National Mycology Reference Laboratory, Republic of Türkiye Ministry of Health, General Directorate of Public Health, Ankara, Turkiye; 13 Department of Infectious Diseases and Clinical Microbiology, Faculty of Medicine, Ankara Yıldırım Beyazıt University, Ankara, Turkiye; 14 Department of Infectious Diseases and Clinical Microbiology, Faculty of Medicine, Health Science University, Sultan 2. Abdülhamid Han Training and Research Hospital, İstanbul, Turkiye; 15 Department of Infectious Diseases and Clinical Microbiology, İstanbul Training and Research Hospital, İstanbul, Turkiye; 16 Division of Intensive Care, Department of Anesthesiology and Reanimation, Gülhane Faculty of Medicine, Health Science University, Etlik City Hospital, Ankara, Turkiye; 17 Department of Infectious Diseases and Clinical Microbiology, Faculty of Medicine, Health Science University, Haydarpaşa Numune Training and Research Hospital, İstanbul, Turkiye; 18 Department of Infectious Diseases and Clinical Microbiology, Faculty of Medicine, Dicle University, Diyarbakır, Turkiye; 19 Department of Infectious Diseases and Clinical Microbiology, Faculty of Medicine, Erciyes University, Kayseri, Turkiye; 20 Department of Anesthesiology and Reanimation, Faculty of Medicine, Yeditepe University, İstanbul, Turkiye; 21 Department of Anesthesiology and Reanimation, Faculty of Medicine, Hacettepe University, Ankara, Turkiye; 22 Department of Infectious Diseases and Clinical Microbiology, Etlik City Hospital, Ankara, Turkiye; 23 Department of Medical Microbiology, Faculty of Medicine, Hacettepe University, Ankara, Turkiye

**Keywords:** *Candida auris*, consensus report, management, infection control, prevention measures, surveillance

## Abstract

*Candida auris* is an emerging fungal pathogen that has become a critical global health concern due to its high antifungal resistance and potential to cause nosocomial outbreaks. Since its initial identification in Japan in 2009, *C*. *auris* has spread rapidly, posing significant treatment challenges across various healthcare settings worldwide. The biofilm formation ability of *C*. *auris* enhances its resilience against disinfectants and antifungal agents, complicating infection control in healthcare environments. This consensus report was developed by a collaboration between several Turkish medical societies including the Turkish Society of Infectious Diseases and Clinical Microbiology Specialty (EKMUD), the Turkish Society of Anesthesiology and Reanimation (TARD), the Turkish Intensive Care Society (TYBD), the Infectious Diseases – International Research Initiative (ID-IRI), the Clinical Microbiology Specialist Society (KLIMUD), the Turkish Microbiology Society (TMC), and the Public Health Institution of Türkiye (PHIT) under the Ministry of Health. The report provides a comprehensive overview of *C*. *auris* and its management, with a focus on the epidemiology, antifungal resistance mechanisms, recommendations for diagnostic and therapeutic challenges, infection control and prevention measures, and surveillance of *C*. *auris*. This consensus report aims to establish standardized diagnostic protocols, improve national surveillance systems, and promote effective infection control measures to mitigate *C*. *auris*-related health risks in Türkiye. It also offers comprehensive national recommendations and addresses the need for interinstitutional collaboration, improve public health, and strengthen the healthcare response to this pathogen.

## 1. AIMS AND SCOPE

Türkiye lies at the intersection of Europe, Asia, and Africa. This geographic position has not only led to considerable economic and cultural influences over the centuries but has also contributed to the epidemiology of various infectious diseases that emerge across the country [1]. *Candida auris* is a type of fungus that can cause deadly infections and has become a significant health issue in recent years both in Türkiye and worldwide, capable of causing outbreaks in hospitals. This consensus report has been produced to inform healthcare professionals about the epidemiology, antifungal resistance profiles, diagnosis, and therapeutic courses of *C*. *auris*. The goal is to help healthcare professionals to determine effective empirical treatment approaches based on national antifungal resistance profiles, to raise awareness regarding interinstitutional collaboration, to develop comprehensive national health strategies regarding this microorganism, to identify and standardize the necessary laboratory methods for the accurate diagnosis of *C*. *auris*, and to develop national health policies to combat *C*. *auris*. The societies, institutions, and groups contributing to this consensus report include the Turkish Society of Infectious Diseases and Clinical Microbiology Specialty (EKMUD), the Turkish Society of Anesthesiology and Reanimation (TARD), the Turkish Intensive Care Society (TYBD), the Infectious Diseases – International Research Initiative (ID-IRI), the Clinical Microbiology Specialist Society (KLIMUD), the Turkish Microbiology Society (TMC), and the Public Health Institution of Türkiye (PHIT) under the Ministry of Health of the Republic of Türkiye.

## 2. EPIDEMIOLOGY

*C*. *auris* was first isolated and identified in 2009 from a case of external otitis in Japan and began to attract attention with the emergence of nosocomial clonal outbreaks in India. Today, it causes infections and outbreaks in healthcare facilities and presents treatment challenges due to antifungal resistance [2–7]. In 2022, *C*. *auris* was included in the World Health Organization’s (WHO) documented priority list of fungal pathogens, highlighting its critical importance^1^. *Candidozyma* is the newly proposed genus classification for *C*. *auris* and related species. [8].

The global spread and increasing incidence of *C*. *auris* have led to its prioritization in scientific research. Since its identification in 2009, there have been a total of 907 original research articles published related to *C*. *auris*. This means that *C*. *auris* will continue to be an important area of research, particularly for understanding antifungal resistance and resistance epidemiology in the coming years [9,10].

### 2.1. Global epidemiology

*C*. *auris* has six identified clades to date. Clade I (South Asia), Clade II (East Asia), Clade III (Africa), Clade IV (South America), Clade V (Iran), and Clade VI (Singapore-Bangladesh) have been defined [11,12]. Clades I, III, and IV are primarily responsible for invasive infections, nosocomial transmission, and large-scale hospital outbreaks while it is noteworthy that Clade II has only been isolated from ear infections so far and is more susceptible to antifungal drugs [2,12].

Although the reason for the simultaneous emergence of *C*. *auris* in different regions of the world is unknown, it appears that new or increased antifungal selection pressure on humans, animals, and the environment may play a role. To understand the global spread and epidemiology of *C*. *auris*, whole-genome sequencing analyses were conducted on 54 *C*. *auris* strains isolated in Pakistan, India, South Africa, and Venezuela between 2012 and 2015, as well as the first Japanese isolate. The strains were grouped into clades according to geographic region, and each clade showed clonal characteristics [13].

According to data from the Centers for Disease Control and Prevention (CDC), the number of *C*. *auris* infections in the U.S. rose from 476 clinical cases in 2019 to 1471 cases in 2021 [14]. Data from the European Centre for Disease Prevention and Control (ECDC) also indicate an increase in *C*. *auris* infection cases and outbreaks in Europe [15]. There are publications that delve into the epidemiology of *C*. *auris* infections with detailed assessments for different continents [2,16]. Following the first isolation of *C*. *auris* in Japan in 2009, various *C*. *auris* infections, including candidemia, have been reported from Asian countries. Retrospective investigations have identified the first *C*. *auris* candidemia isolate from a 1-year-old child in South Korea in 1996, who was diagnosed with hypoxic encephalopathy and aspiration pneumonia [2]. Cases of *C*. *auris* infection have been published from six African countries, mostly from South Africa [16]. *C*. *auris* was identified as the third most common cause of candidemia in South Africa [17]. Molecular epidemiological studies of isolates from the African continent found that the majority of 127 African isolates were classified as Clade III (78.7%), followed by Clade I (14.2%), Clade IV (6.3%), and Clade II (0.8%) [16].

Due to limited surveillance studies and laboratory capabilities necessary for identification, the available data on the prevalence of *C*. *auris* worldwide likely do not reflect the true numbers [17]. *C*. *auris* infections have been reported from over 40 countries across six continents, including the U.S., Germany, Austria, Belgium, the United Arab Emirates, China, Denmark, Finland, France, South Africa, South Korea, India, the Netherlands, the United Kingdom, Ireland, Spain, Israel, Sweden, Switzerland, Italy, Canada, Kenya, Colombia, Kuwait, Malaysia, Norway, Pakistan, Panama, Poland, Russia, Saudi Arabia, Türkiye, Oman, Venezuela, and Greece [18–20].

*C*. *auris* is known to cause outbreaks, particularly in intensive care units (ICUs) [21]. *C*. *auris* cases and outbreaks were identified during the COVID-19 pandemic due to the pressure on healthcare systems and the challenges in implementing adequate infection control measures and institutional antibiotic stewardship programs [18]. An outbreak that emerged in a center in Spain is considered the largest clonal outbreak in Europe [21]. The UK and Italy are among the European countries that have reported nosocomial outbreaks related to *C*. *auris* [18].

### 2.2. National epidemiology

It is not possible to obtain precise figures on *C*. *auris* prevalence in Türkiye due to the lack of mandatory reporting of infections before September 10, 2024, and the absence of surveillance data. In certain tertiary hospitals in Türkiye, identification of *C*. *auris* strains, antifungal resistance determination using the reference microdilution method, and advanced molecular investigations are performed. The instiutions that struggle to conduct these tests voluntarily send strains to the National Mycology Reference Laboratory (NMRL)-PHIT.

When the current literature is examined from a national epidemiological perspective, the first case of *C*. *auris* fungemia was reported in İstanbul in 2021 [22] and was followed by other cases [23]. During the COVID-19 pandemic, a Türkiye-based study retrospectively evaluated candidemia cases over a 17-month period between 2020 and 2021, finding that *C*. *auris* was the causative agent in 9% of candidemia cases [24]. Additionally, cases from Türkiye have been included in international multicenter studies [25–27].

The first seven *C*. *auris* infections reported in Türkiye were identified and examined for biochemical profiles and suspectability to antifungal drugs. The strains showed characteristics consistent with Clade I [28]. In a nosocomial outbreak that occurred in a COVID-19 ICU, 10 cases were identified and the *C*. *auris* strains responsible belonged to Clade I [29]. The analyses that report the phylogenetic characteristics confirmed by whole-genome sequencing isolated in Türkiye are qualitative and currently encompass a limited number of strains.

### 2.3. Antifungal resistance

#### 2.3.1. Global antifungal resistance

In a systematic review addressing *C*. *auris* and its infections, in vitro antifungal resistance results obtained from 25 published studies were evaluated. Fluconazole resistance rates were high in these studies, ranging from 87% to 100%. The susceptibility rates to other azoles varied across the studies. Although some studies reported resistance rates for amphotericin B as low as 0% and as high as 58–61%, most studies showed resistance rates ranging from 8% to 35%. Echinocandins resistance rates were low, ranging from 0% to 8% [21]. Recently identified Clade VI isolates have shown lower minimum inhibitory concentration (MIC) values for fluconazole, placing them in the susceptible category [11].

#### 2.3.2. Antifungal resistance in Türkiye

High MIC levels against fluconazole were detected using the broth microdilution method for all four cases reported in two different hospitals in Türkiye [22,23]. The antifungal susceptibility results of one isolate, determined using three different susceptibility methods (microdilution, gradient strip, and colorimetric microdilution) in the same study, were not consistent with the reference method [22]. During the same period, susceptibility tests conducted using the gradient strip method on an isolate from a SARS-CoV-2 positive *C*. *auris* fungemia patient from a different hospital reported resistance to fluconazole and amphotericin B, while showing susceptibility to caspofungin and anidulafungin [30]. In August 2021, another hospital reported resistance to azoles and caspofungin using the colorimetric antifungal susceptibility method in one patient [31]. In a study conducted in August 2023 using colorimetric microdilution, a strain resistant to azoles, amphotericin B, and caspofungin was identified; however, whole-genome sequencing did not reveal any genomic data specific to resistance against amphotericin B and caspofungin [5]. During the COVID-19 pandemic, between June 2020 and August 2022, the NMRL-PHIT reported that fluconazole resistance was observed in all 59 *C*. *auris* isolates, although panfungal resistance was not detected^2^.

Based on data up to August 2023, although most *C*. *auris* isolates analyzed in NMRL-PHIT showed high fluconazole MIC values (≥256 mg/mL), the MIC values for other azoles were variable. Echinocandins were identified as the most sensitive antifungals, followed by amphotericin B and azoles, respectively (Table 1)^3^.

## 3. MICROBIOLOGY

### 3.1. Microbiological characteristics of *C. auris*

*C*. *auris* is a *Candida* species that does not form germ tubes, is negative for raffinose, and is positive for sucrose, trehalose, and glucose, while being sensitive to cycloheximide. It can easily grow on routine isolation media such as blood agar and chocolate agar, as well as on mycological media like Sabouraud dextrose agar and *Candida* chromogenic agar. It can grow at temperatures ranging from 30 to 42 °C [32]. In addition to being thermotolerant, it also has halotolerant properties. On Sabouraud dextrose agar, it forms white, cream-colored, smooth, and undulate colonies. On various commercial chromogenic media, the colonies may appear pale purple, light pink (CHROMagar *Candida*, Paris, France), or blue (CHROMagar *Candida* Plus, Paris, France). Growth is inhibited in the presence of cycloheximide [33,34].

Microscopic examination reveals oval or elongate budding cells measuring 2.0–3.0 × 2.5–5.0 μm, appearing singly, in pairs, or in clusters. However, under stress conditions such as high salt concentration, the formation of pseudohyphae has also been observed [35]. On cornmeal Tween 80 agar, it appears as budding yeast cells. Pseudohyphal formation is either absent or extremely rare, and true hyphal formation is not observed [33].

### 3.2. Biofilm formation capacity

Biofilm formation is one of the most important pathogenic characteristics of *C*. *auris*. It plays a significant role in the colonization of microorganisms in hospital environments and within the human body, as well as in resistance to disinfectants and antifungal drugs. *C*. *auris* is divided into two groups based on aggregation characteristics. An aggregate is a large cluster of cells that forms when daughter cells do not separate from the mother cell after budding. These strains have a lower capacity for biofilm formation [36,37].

In standard laboratory settings, *C*. *auris* forms less biofilm compared to *Candida albicans* in terms of biomass and metabolic activity. The absence or rarity of pseudohyphae formation leads to a reduction in the ability to adhere to surfaces and form biofilms [36,37]. However, in synthetic media designed to mimic axillary skin conditions in vitro and in excised porcine skin (ex vivo), *C*. *auris* can form multilayered biofilms containing ten times more cell load compared to *C*. *albicans* [38]. The *C*. *auris* biofilm is more resilient to stress conditions such as high salt concentration, fatty acids, and desiccation compared to *C*. *albicans*. This characteristic contributes to the status of *C*. *auris* as a significant environmental pathogen [39].

### 3.3. Antifungal resistance mechanisms

The development of antifungal resistance in *C*. *auris* occurs through mechanisms such as changes in drug targets, overexpression of drug targets, alterations in drug uptake and expulsion processes, and biofilm formation [40].

#### 3.3.1. Resistance to azole antifungal drugs

Mutations occurring in the hotspot regions of the *ERG11* gene, which encodes the lanosterol 14-alpha-demetylase enzyme responsible for the conversion of lanosterol to ergosterol, play a significant role in the development antifungal drug resistance (F126L, Y132F, and K143R). These mutations cause structural changes in the drug target, resulting in a decreased binding affinity for the drug [13,41–43]. Another mechanism is the overexpression of ERG11. The increased production of the lanosterol 14-alpha-demetylase enzyme overcomes the inhibitory effect of antifungals on the enzyme, allowing for continued active synthesis despite treatment [41].

Efflux pumps are responsible for the expulsion of toxic molecules, such as azole antifungals. In *C*. *auris*, there are two types of efflux pumps associated with drug resistance: ATP-binding cassette (ABC) transporters and the major facilitator superfamily (MFS) [41]. The overexpression of the genes coding for efflux pumps (*MDR*, *CDR1*) is one of the most significant resistance mechanisms against azole antifungal drugs in *Candida* spp. [41,44].

#### 3.3.2. Echinocandin resistance

Mutations in the *FKS1* gene (S639P, S639F, S639Y, F635C, S635P, and S635T) contribute to echinocandin resistance against antifungal drugs [42,43,45]. The *FKS1* and *FKS2* genes are two components of 1,3-β-D-glucan synthase and encode 1,3-β-D-glucan—an important part of the fungal cell wall. This enzyme is the target of echinocandins [46].

#### 3.3.3. Amfoterisin B resistance

Amphotericin B-resistant strains of *C*. *auris* have been reported; however, the resistance mechanisms have not been sufficiently elucidated [47]. A mutation in the *ERG6* gene for sterol methyltransferase in *C*. *auris* strains can cause resistance to amphotericin B by disrupting ergosterol synthesis [48].

#### 3.3.4. Antifungal resistance and biofilm

*C*. *auris* biofilms contribute to increased resistance to antifungal drugs. Various factors such as the density of fungal cells within the biofilm structure, communication through signaling molecules, and the activity of efflux pumps, cell persistence, extracellular matrix, and the overexpression of drug targets all contribute to antifungal resistance [49].

### 3.4. Microbiological diagnosis and diagnostic challenges

Phenotypic methods used species identification in the *Candida* genus are inadequate for identifying *C*. *auris*. Yeasts are named at the species level based on their phenotypic characteristics, such as the formation of germ tubes within 2 h in serum, their morphology on cornmeal-Tween 80 agar, carbohydrate assimilation profiles, reproduction times, and urease production. In addition, yeast fungi can also be identified using automated identification systems. However, when all these phenotypic identification methods are used, the *C*. *auris* species may be misidentified as *Candida haemulonii*, *Candida duobushaemulonii*, *Rhodotorula glutinis*, *Candida sake*, *Candida intermedia*, *Saccharomyces kluyveri*, *Candida catenulata*, *Candida famata*, *Candida guilliermondii*, *Candida lusitaniae*, or *Candida parapsilosis*. The most reliable phenotypic method for species-level identification is matrix-assisted laser desorption ionization time-of-flight mass spectrometry (MALDI-TOF MS). The use of this proteomics-based technique in routine microbiology laboratories is recommended [50,51].

### 3.5. Antifungal susceptibility testing

#### 3.5.1. Antifungal susceptibility method

Susceptibility testing for amphotericin B, azoles, and echinocandins should be conducted using the reference broth microdilution method (Clinical and Laboratory Standards Institute (CLSI M27-A4) protocol) for *Candida* spp. [52] and the results should be interpreted according to the new species-specific resistance breakpoints (CLSI M60) [53].

#### 3.5.2. Antifungal resistance breakpoints

Specific clinical resistance breakpoints (CRBP) for *C*. *auris* have not yet been established by the Clinical and Laboratory Standards Institute (CLSI) and the European Committee on Antimicrobial Susceptibility Testing (EUCAST). Based on data collected on *Candida* spp. and numerous expert opinions, the CDC has provided guidance on temporary breakpoints for interpreting the MIC values of *C*. *auris*. These temporary breakpoints established by the CDC are used for the assessment of sensitive (S) and resistant (R) strains for anidulafungin, caspofungin, amphotericin B, and fluconazole. However, MIC breakpoints for voriconazole and itraconazole against *C*. *auris* have not been established in the CDC guideline document^4^ [54].

#### 3.5.3. Standard strains

According to CLSI recommendations, the standard strains for internal quality control tests of antifungal susceptibility using broth microdilution (CLSI M27-A4) are *Candida krusei* ATCC 6258 and *C*. *parapsilosis* ATCC 22019.

### 3.6. Molecular identification methods

The challenges in the phenotypic identification of *C*. *auris* necessitate the use of molecular methods. Polymerase chain reaction (PCR) techniques using species-specific primers may be preferred for molecular identification. DNA sequence analysis for species verification can be conducted using the internal transcribed spacer (ITS) and/or 28S ribosomal DNA (rDNA) D1/D2 regions, recognized as barcode genes for fungi. Additionally, whole-genome analysis is available for genotyping strains and determining subspecies. Establishing clades may also be necessary in epidemiological studies. In routine laboratory practices, accurate species-level identification and susceptibility testing are sufficient, thereby facilitating patient isolation and management of treatment processes [11,51,55–57].

### 3.7. Microbiological screening policy

*C*. *auris* is a unique species of *Candida* that can colonize the skin and survive for extended periods on inanimate surfaces. There are differing opinions regarding the screening of patients and surfaces in healthcare facilities for *C*. *auris*. Swab samples should be taken from the skin be inoculated onto chromogenic media and subjected to PCR for the identification of the yeast. Studies have shown that culture and PCR methods are equally specific and sensitive. The macroscopic appearance of *C*. *auris* colonies on nonchromogenic media is similar to that of other *Candida* species. On the other hand, the chomogenic medium CHROM agar *Candida* Plus has been successfully used to demonstrate *C*. *auris* colonization. Evidence indicates that screening patients for colonization and calculating colonization indices during interinstitutional patient transfers can help prevent outbreaks. For screening, swab samples can be taken from the nose, throat, rectum, axilla, and groin [25]. However, we particularly recommend taking swab samples from the nose, axilla, and groin. A single swab can be used to collect samples from both the axilla and groin. The isolation of *C*. *auris* from more than half of the sampled areas indicates a high colonization index. A high index suggests that the patient is at risk of invasive infection and should be monitored [58,59].

### 3.8. Reference laboratory infrastructure in the country

The national reference laboratory capacity has been expanded to include molecular testing to cope with the increase in *C*. *auris* case reports in Europe and requests for the diagnosis of invasive fungal infections and antifungal susceptibility testing in Turkish hospitals. Since 2015, the General Directorate of Public Health NMRL-PHIT has been accredited according to International Organization for Standardization (ISO) 15189 standards for yeast and mold identification using reference methods and for antifungal susceptibility testing using the microdilution method.

### 3.9. National diagnostic policy recommendations for *C. auris*

Outbreaks of this highly pathogenic yeast in health care institutions can be prevented and contained by rapidly and accurately identifying patients infected or colonized with *C*. *auris*, quickly determining susceptibility profiles, and effectively implementing infection control measures^5^ [60].

Any *Candida* spp. isolate associated with invasive infections and/or screening samples taken from long-term ICU patients who have encountered difficulties in treatment, should be identified at the species level. If suspicious *Candida* spp. are detected, additional studies should be conducted to confirm that they are not *C*. *auris*. These studies should include MALDI-TOF Biotyper analysis with a library known to already contain *C*. *auris* or sequencing analysis of the rRNA, D1/D2 region, and *ITS1* and *ITS2* gene regions. These facilities are available at NMRL-PHIT.

#### 3.9.1. Submission of isolates to NMRL-PHIT for identification

Since June 2020, during the COVID-19 pandemic, patient samples with a preliminary diagnosis of *C*. *auris* have been sent to NMRL-PHIT from the ICUs of various hospitals [23]. Pure culture isolates should be sent to NMRL-PHIT on Sabouraud agar along with the appropriate form. According to the NMRL diagnostic algorithm for the identification of *C*. *auris*, after inoculation on chromogenic media, morphological examination, and biochemical tests, identification is carried out using MALDI-TOF MS (Bruker Daltonics, Germany), followed by sequencing analysis of the rRNA, large subunit rRNA (D1/D2 region), and *ITS1* and *ITS2* gene regions.

Given the increasing prevalance of this pathogen and its role in causing outbreaks in hospitals, obtaining epidemiological data including sequencing analysis and resistance profiles are of great importance.

## 4. *C*. *auris* COLONIZATION

### 4.1. Colonization in patients

*C*. *auris* can colonize different anatomical areas in the human body in addition to being a causative agent of invasive infections [61]. This poses challenges in controlling hospital-acquired outbreaks by facilitating the spread to the patient environment and equipment. *C*. *auris* commonly colonizes the skin, axilla, groin, mucosal surfaces of the gastrointestinal and genitourinary systems, as well as the respiratory tract (oropharynx, nose) and external ear. It can also colonize around central venous catheters and tracheostomy sites [2,62,63].

It is generally accepted that colonization can occur at least 4 h after contact with an infected person or surface, and that invasive infections may develop approximately 48 h after hospital admission [63–65].

In geographic regions with high *C*. *auris* incidence, colonization rates have been reported to vary between 2.5% and 33.9% [66,67]. The risk factors for *C*. *auris* colonization are as follows [68,69]:

- Prolonged hospitalization and stay in intensive care
- Prolonged use of broad-spectrum antibiotics
- Frequent use of invasive devices
- Inadequate surveillance

Colonization in patients can last for extended periods. In certain individuals who remain hospitalized, colonization can exceed 1 year. There have also been reports of carriers still harboring the organism eight months following their discharge from the hospital [70,71].

### 4.2. Surface colonization

*C*. *auris* is capable of colonizing surfaces within the patient environment as well as the patients themselves. Research has shown a significant association between the environmental contamination of *C*. *auris* and the number of body areas colonized in patients [72,73]. Unlike other *Candida* species that typically colonize, *C*. *auris* primarily colonizes the skin [71]. Additionally, *C*. *auris* can be reisolated from the surroundings of infected or colonized patients 4 h postcleaning and disinfection. In vitro studies indicate that *C*. *auris* can remain viable on both dry and moist surfaces for a duration of 7 to 28 days [74,75].

## 5. *C*. *auris* INFECTIONS

### 5.1. Risk factors facilitating the development of C. auris infections

The first stage in the development of invasive infections in patients is colonization [64,76]. The literature reports that among individuals colonized with *C*. *auris*, 17% experienced one or more episodes of candidemia [77], and approximately 10% developed other invasive infections [68]. The nosocomial transmission of *C*. *auris* is particularly associated with intense invasive procedures performed during hospital admission, as well as with diseases that require close monitoring, and comorbidities [63,78,79].

The identified risk factors that increase susceptibility to *C*. *auris* infections are similar to those associated with other *Candida* species. These factors include [77,78,80–82]:

- Hematopoietic stem cell transplantation
- Solid organ transplantation
- Use of glucocorticoids
- Neutropenia
- Prolonged hospitalization (particularly in ICUs)
- Mechanical ventilation
- Central venous catheters
- Urinary catheters
- Long-term use of broad-spectrum antibiotics and antifungal treatments
- Underlying conditions (lung disease, malignancy, chronic kidney disease, diabetes mellitus)
- Vascular surgery or surgeries performed within the last 30 days

In ICUs, the incidence of candidemia after colonization with *C*. *auris* has been reported to be 17% by the 30th day and 25% by the 60th day [77]. Mechanical ventilation presents a unique situation; clinical settings that provide mechanical ventilator support have been observed to have 10 times higher rates of *C*. *auris* colonization compared to those that do not. In a study conducted in nursing homes, the use of carbapenems and fluconazole within the past 90 days has been identified as an extra risk factor in facilities that provide ventilator support [68].

*C*. *auris* can affect at risk populations across all age groups. In children, nosocomial infections predominantly occur in those under 1 year of age, particularly in the presence of coexisting conditions such as prematurity and malignancy, as well as risk factors like intravenous catheter use, parenteral nutrition, and mechanical ventilation [83].

### 5.2. Infections caused by *C. auris*

*C*. *auris* can lead to serious, life-threatening superficial skin infections and bloodstream infections. The clinical signs and symptoms are similar to those of other *Candida* infections^6^. While *C*. *albicans* is more commonly associated with mucosal infections (such as oropharyngeal and vulvovaginal infections), *C*. *auris* is more frequently responsible for bloodstream infections, urinary tract infections, and wound infections [84].

Colonization can develop shortly after contact, and invasive infections may occur days to months following colonization^7^ [61]. The overgrowth of *C*. *auris* on mucocutaneous surfaces can facilitate the development of bloodstream infection, particularly when there is poor adherence to aseptic techniques during invasive catheter applications [85]. Moreover, the literature reports cases of *C*. *auris* related vulvovaginitis, complicated intraabdominal infections, surgical site infections, skin abscesses related to catheter insertion sites, burn infections, otitis, pericarditis, myocarditis, osteomyelitis, and meningitis [85–88]. When *C*. *auris* is detected in nonsterile body sites, it is essential to assess whether the patient exhibits clinical symptoms to distinguish between colonization and infection^7^.

*C*. *auris* infections are often seen in immunosuppressed individuals with underlying conditions who have been hospitalized for extended periods and have undergone invasive procedures^8^. However, they can also occur in immunocompetent hosts. Due to their resistant nature, potential for invasive infections, association with hospital outbreaks, and high morbidity and mortality rates, early diagnosis and treatment are essential.

### 5.3. Prognosis of *C. auris* infections

*C*. *auris* can cause hematogenous spread, leading to multiorgan failure. Additionally, bloodstream infections that develop secondarily from a localized infection can result in sepsis and multiple organ failure involving the kidneys, heart, lungs, eyes, brain, liver, and spleen, ultimately leading to death^9^. It has also been reported that in cases of *C*. *auris* candidemia, persistent positive blood cultures over five days can lead to recurrent candidemia episodes [61].

The overall case-fatality rate associated with *C*. *auris* infections has been reported to reach 40%–60% in the literature [89–91]. In the analysis of *C*. *auris* cases, comorbidities, particularly kidney disease, have been recognized as important risk factors for mortality among infected patients [92]. Another study identified lung and central nervous system comorbidities as additional risk factors for death [93]. Prognosis is influenced by various factors, including the patient’s age, severity and extent of the underlying infection, virulence of the pathogen, antifungal resistance, and the timely initiation of appropriate antifungal therapy^9^ [94]. One study reported an overall case-fatality rate of 39%, with a rate of 45% in bloodstream infections [95]. In another study that assessed mortality indicators, the presence of heart failure, kidney failure, hemodialysis, central venous catheters, invasive ventilation, and total parenteral nutrition significantly correlated with mortality. Conversely, the initiation of echinocandin treatment, removal of catheters (if present), wound debridement, and source control have been reported as factors that positively influence patient outcomes [25]. However, determining the extent to which death can be directly attributed to *C*. *auris* in this high-risk patient population is challenging.

The mortality rate of *C*. *auris* infections reported in Europe is around 20%, which is lower compared to 44% reported in Asia [95]. The lower mortality rate in Europe may be attributed to more effective surveillance and rational treatment courses in developed countries, as well as the potential for detecting more nonfungemia *C*. *auris* infections, the presence of different clades, and heterogeneity between the studies [95].

## 6. TREATMENT

### 6.1. Antifungal treatment

Considering the epidemiological data in Türkiye, echinocandin therapy should be the first choice for patients with *C*. *auris* infections. Particularly for patients who have undergone *C*. *auris* species identification but are awaiting antifungal susceptibility results, echinocandins should be the preferred empirical treatment. Standard dosing includes: a loading dose of 70 mg IV for caspofungin, followed by a daily maintenance dose of 50 mg IV; for micafungin, a daily dose of 100 mg IV; and for anidulafungin, a loading dose of 200 mg IV, followed by a maintenance dose of 100 mg IV [96]. Additionally, antifungal susceptibility testing should be performed on all *C*. *auris* isolates, and the results should be used to provide individualized antifungal treatment choices.

Patients receiving antifungal therapy should be closely monitored with follow-up blood cultures. If a patient does not respond clinically to echinocandin therapy or if fungemia persists for five days, switching to or adding liposomal amphotericin B (5 mg/kg daily) may be considered [97]. For patients who continue to test positive for *C*. *auris* in blood cultures, the possibility of infective endocarditis, hepatosplenic candidiasis, inadequate source control, and resistance development during treatment should be considered. Consequently, echocardiography should be conducted for suspected infective endocarditis, abdominal imaging for suspected hepatosplenic candidiasis, and central venous catheters should be promptly removed in patients with catheter-related infections. When antifungal resistance development is suspected, antifungal susceptibility testing should be performed on repeat cultures [96–98].

Routine fundoscopic examinations should be conducted for patients with *C*. *auris* candidemia. In neutropenic patients, since the signs of choroidal and vitreous infections are minimal, ophthalmological examination should be postponed until at least the first week after the resolution of neutropenia [96]. Although the incidence of endophthalmitis in candidemia patients is low, treatment failure may occur due to insufficient concentrations of systemically administered echinocandins reaching the vitreous. Patients with chorioretinitis can be treated with a systemic antifungal agent in the absence of macular or vitreous involvement. If there is macular involvement or endophthalmitis, the injection of voriconazole or amphotericin B intravenoulsy is recommended, provided the organism is susceptible. As a systemic antifungal, voriconazole is preferred; if the strain is resistant to voriconazole, a combination of liposomal amphotericin B and flucytosine is suggested [96].

### 6.2. Treatment duration

Patients with candidemia and no localized infections, such as infective endocarditis or endophthalmitis, should receive at least 2 weeks of treatment after blood cultures return negative. This is the same for candidemia caused by other species. Additionally, symptoms attributed to candidemia and neutropenia should be resolved before discontinuing antifungal treatment [96]. In patients with infective endocarditis, endophthalmitis, hepatosplenic candidiasis, or bone or joint involvement, a longer treatment course should be administered, even though the exact duration is not clearly defined.

### 6.3. New antifungals

*C*. *auris* is a multidrug resistant fungus that can cause serious infections in hospitals and other healthcare settings. The detection of antifungal resistance, even to the primary treatment option echinocandins, highlights the need for new therapeutic alternatives. New antifungal agents may include well-known classes (such as azoles, polyenes, or beta-D-glucan synthase inhibitors) or compounds with entirely new mechanisms of action. The classification of these new antifungals based on their mechanisms of action is illustrated in Figure and their efficacy in fungal infections is presented in Table 2 [99].

Three novel antifungal agents have been recognized as effective against *C*. *auris*. These include rezafungin, ibrexafungerp, and fosmanogepix. In vitro susceptibility testing indicates that manogepix/fosmanogepix (MIC90 = 0.03 mg/L), ibrexafungerp (MIC90 = 1 mg/L), and rezafungin (MIC mode = 0.25 mg/L) exhibit effectiveness. Furthermore, these drugs have shown efficacy in both in vitro and in vivo studies against *C*. *auris* strains that are resistant to echinocandins [100].

#### 6.3.1. Rezafungin

Rezafungin is a second-generation echinocandin, developed by the modification of anidulafungin. The long half-life of the drug (130 h) allows the convenience of once-weekly dosing. Its activity spectrum is comparable to that of other echinocandins, including coverage for *C*. *auris*. In a comparative study involving adult patients with candidemia, rezafungin was found to demonstrate equivalent efficacy to caspofungin and has received U.S. Food and Drug Administration (FDA) approval for this use [101].

#### 6.3.2. Ibreksafungerp

Ibrexafungerp is a semisynthetic derivative of enfumafungin and a noncompetitive inhibitor of the 1,3-β-D-glucan synthase enzyme complex. While it is not chemically classified as an echinocandin, its mechanism of action is similar to that of echinocandins. It also shows some degree of efficacy against echinocandin-resistant *Candida* isolates. It can be administered orally and shows high penetration in intraabdominal lesions. Ibrexafungerp has been approved for the treatment of vulvovaginitis and is currently under evaluation for the treatment of nonneutropenic invasive candidiasis, invasive fungal infections resistant to other treatments, and *C*. *auris* infections [102].

#### 6.3.3. Fosmanogepix

Fosmanogepix is a new drug with broad-spectrum activity that targets an enzyme in the glycosylphosphatidylinositol biosynthesis pathway. In a phase 2 study of fosmanogepix, subgroup analysis of nine intensive care patients with *C*. *auris* candidemia showed treatment success and survival in eight cases (89%). A phase 3 study comparing fosmanogepix to a combination of caspofungin and fluconazole in patients with candidemia and/or invasive candidiasis is ongoing [103].

While the development of new antifungals increases our treatment options, the presence of organisms like *C*. *auris* that are resistant to antifungals and have a high rate of nosocomial spread remains a significant issue. Rational antifungal use and infection control are crucial for protecting existing and developing antifungals.

### 6.4. Antifungal stewardship

The challenge of resistance faced by physicians regarding *Candida* species has historically been confined to naturally acquired fluconazole resistance. However, the swift global proliferation of *C*. *auris* has underscored the need for meticulous implementation of antifungal stewardship (AFS) programs. The main aim of AFS is to deliver treatment in the most effective and precise manner while reducing the adverse effects associated with antifungal use (such as toxicity, side effects, drug interactions, and the emergence of resistance) [104]. As a fundamental principle, the 5 Ds rule of antibiotic stewardship is relevant in this context as well:

- D (Drug): The correct antifungal medication
- D (Deescalation): Narrowing the spectrum, transitioning from parenteral to oral treatment
- D (Discontinuation of therapy): Stopping treatment after the appropriate duration
- D (Dose): Correct dosing
- D (Diagnosis): Accurate diagnosis

Timely and precise identification of fungi with resistance issues, such as *C*. *auris*, is essential for quickly determining the resistance profile and for the prompt implementation of appropriate therapy and infection control measures [105,106].

Within a healthcare institution, both restriction (coercive) and persuasion (voluntary) strategies can be utilized together in the implementation of AFS. The most recognized restriction method involves requiring approval for antibiotic use. In Türkiye, all parenteral antifungal drugs can only be reimbursed with the approval of an infectious disease specialist, except for oral fluconazole. Thus, a separate institutional-level restriction is unnecessary. It is crucial to ensure the effective functioning of AFS principles. The infectious disease specialist should not simply fulfill a role of approving on paper or electronically; they must evaluate the patient in person and actively engage as part of the team from the initiation to the conclusion of the treatment process.

Among persuasion techniques, monitoring and feedback is crucial. In hospitals, while antifungal treatment is managed by infectious disease specialists, monitoring and providing feedback on treatments proposed by various infectious disease experts can help identify any overlooked errors [107].

The performance of the AFS program must be evaluated (Table 3) [104]. Infectious disease physicians are the driving force in establishing the necessary infrastructure for systematic measurement of these metrics. Just like in infection control practices, the support, assistance, and involvement of hospital management should be ensured in AFS as well.

The AFS program cannot be effectively implemented or managed by just one person. Like antibiotic stewardship, it should be conducted by a team that includes hospital management, clinical pharmacists, infection control team representatives, a quality team representative, microbiology laboratory staff, pharmacy managers, and an information technology representative in facilities where data is electronically maintained. The same team can function as the antibiotic stewardship team, eliminating the need for a separate structure. The team should meet at regular intervals to identify problems, design and implement solutions, and evaluate whether improvements have been made by analyzing the outcomes.

## 7. INFECTION CONTROL AND PREVENTION

### 7.1. Hand hygiene

When providing care for patients with *C*. *auris*, healthcare providers must adhere to standard hand hygiene protocols. An alcohol-based hand sanitizer is recommended for use with *C*. *auris* when hands are not visibly dirty. If hands are visibly soiled, they should be washed thoroughly with soap and water. It is important to note that wearing gloves does not substitute for proper hand hygiene.

### 7.2. Patient management

#### 7.2.1. Isolation and cohorting

The effective management of *C*. *auris* spread heavily relies on infection control practices. Consequently, all patients who are colonized or infected with *C*. *auris* must be placed in contact isolation^10^ [108]. Whenever possible, these patients should be isolated in single rooms. If the availability of single rooms is limited, it is advisable to prioritize those patients who pose a higher risk of transmission, such as individuals with uncontrolled secretions, diarrhea, or draining wounds [62,109]. When single rooms are unavailable, patients colonized with *C*. *auris* may be housed together in the same room. Grouping patients colonized with *C*. *auris* into a designated unit or section within hospitals helps prevent further spread by reducing the movement of healthcare staff and equipment into noncolonized areas. Furthermore, healthcare personnel assigned to care for patients colonized with *C*. *auris* should focus exclusively on these patients for the duration of their shifts [110].

When patients are placed in shared rooms, the following strategies should be implemented to minimize transmission in the hospital. These strategies are applicable to all shared rooms, regardless of the patient’s colonization or infection status^11^ [60,110]:

- Maintain a minimum distance of 1 m between beds
- Use privacy curtains to limit direct interaction
- Clean each bed and its surrounding furniture as if they were in separate rooms
- Clean and disinfect shared or reusable equipment before each use
- Change mop heads, cleaning cloths, and other cleaning tools when transitioning to different patient areas
- Increase the frequency of cleaning and disinfecting environmental surfaces
- Ensure that all healthcare staff entering the room are properly equipped with personal protective equipment
- Perform hand hygiene before and after every patient interaction
- Limit patients’ contact with visitors [111,112]

#### 7.2.2. Screening of patients

Screening of patients who are at high risk of *C*. *auris* colonization is recommended. The following patients should be screened:

1. Individuals who have an epidemiological connection with a patient infected or colonized with *C*. *auris* should be screened through point prevalence surveys. An epidemiological connection includes:
  - Sharing the same room, unit, or other care areas with a patient who has *C*. *auris*
    (even if that person has been discharged),
  - Receiving care from the same healthcare personnel as someone with *C*. *auris* during the same time period
  - Exposure to shared mobile medical equipment used by a patient with *C*. *auris*, especially when there are concerns about adequate cleaning and disinfection
2. Patients who are transferred from another hospital or healthcare facility
3. Routine screening of healthcare workers and environmental sampling is not recommended [113]

#### 7.2.3. The duration of the measures

Patients in healthcare facilities can remain colonized with *C*. *auris* for months, even after being treated for and recovering from acute infections. More than 50% of patients with one or more negative screening culture results have shown regrowth of *C*. *auris* in subsequent screening cultures. Therefore, it is recommended that contact isolation for these patients, including those who have negative control culture results, continue throughout their hospital stay. Routine screening cultures for colonized patients are not recommended [62,63,66,109,110,114]. In the event that patients who are colonized or infected with *C*. *auris* are readmitted to the hospital, contact isolation should be implemented until the screening results are available. The implementation of an alert button in hospital electronic data systems for the identification of patients infected or colonized with *C*. *auris* would be beneficial for taking the necessary isolation precautions during the repeat hospital admissions. Linking these alert systems to a national network to enhance the control of *C*. *auris* spread should be considered.

#### 7.2.4. Provision of supportive healthcare personnel and equipment

*C*. *auris* spreads from patient to patient through the use of contaminated items and through the direct or indirect contact of the hands of healthcare workers. Contact with contaminated items, close contact with patients, and even previous contacts with cases in the preceding months or past room contacts can be significant causes of colonization. Close contact with patients has been associated with a colonization rate of 12%–21% [75,115].

In order to effectively isolate patients who are colonized or infected with *C*. *auris*, adequate supportive healthcare personnel should be available to facilitate proper cleaning and disinfection of the environment. Whenever feasible, single-use items and equipment (such as thermometers, pulse oximeter probes, and noninvasive blood pressure cuffs) should be utilized [111,116,117].

The most significant contributing factors to transmission are the lack of compliance with hand hygiene and personal protective equipment. Prolonged ICU stays, insufficient training among healthcare workers, and environmental contamination are factors contributing to the spread of *C*. *auris* infections. Therefore, individuals caring for patients infected or colonized with *C*. *auris* must pay attention to hand hygiene using soap and water, alcohol-based hand sanitizers, or chlorhexidine-based hand disinfectants. In addition, medical supplies and equipment used for these patients should not be shared, and single-use personal protective equipment (such as gowns and gloves) should be preferred [111,116,117].

#### 7.2.5. Disinfection procedures

Although there is no standardized cleaning and disinfection procedure defined for *C*. *auris*, some recommendations have been suggested in the literature. Prior to decontamination, it is necessary to remove body fluids and organic matter. Patient rooms and restrooms should be cleaned with effective disinfectants two to three times a day. *C*. *auris* can remain metabolically active on plastic surfaces for at least two weeks and on other surfaces for four weeks [17]. In the event of a patient’s discharge or transfer, terminal cleaning procedures should be implemented, and environmental sampling for *C*. *auris* should be conducted [111,116].

Disinfectants such as hypochlorite at a concentration of 1000 ppm or topical hydrogen peroxide-based disinfectants are recommended [111,117,118]. In addition to routine cleaning, peracetic acid, hydrogen peroxide (H_2_O_2_) at less than 1%, vaporized H_2_O_2_ (VHP), and ultraviolet-C type are other methods that can be used for decontamination. Repeated washings should be performed with 2.5 ppm ozonated water (which can eliminate contamination within 2 days using 30 s cycles every 4 h) for colonized sinks in patient rooms [117,119].

#### 7.2.6. Decolonization procedures

Although protocols and procedures for decolonization have not yet been fully established, the implementation of decolonization procedures is supported. For patients on mechanical ventilation, oral nystatin, 2% chlorhexidine wipes, and 0.2% or 1% chlorhexidine dental gel are recommended for oral care. Patients with a history of colonization or infection with *C*. *auris* should be considered colonized for at least 1 year until cultures taken according to the microbiological screening policy are negative. Healthcare workers in close contact with these patients should also implement strict contact precautions [64].

#### 7.2.7. Prevention of catheter related infections and catheter management

*C*. *auris* infections pose a transmission risk similar to that of infections caused by multidrug-resistant bacteria. Therefore, infection control measures must be strictly implemented in the management of urinary tract infections [120]. Following the isolation of *C*. *auris* in urine, point prevalence screening should be conducted on the patient’s contacts for colonization and potential infection risk.

Source control is essential as the first step in managing patients with *C*. *auris* urinary or central catheter colonization or urinary tract infections. It is crucial to remove urinary catheters or central venous catheters in patients infected with *C*. *auris* for source control [25,121]. Strict adherence to care bundles for central, peripheral, and urinary catheters and proper care of the tracheostomy site are fundamental preventive measures [117]. Using chlorhexidine dressings on all catheter insertion sites reduces bloodstream infections [64]. In patients colonized with *C*. *auris*, it is recommended to perform good skin cleaning before the placement of devices and to administer a single dose of antifungal medication in accordance with the antibiogram in the presence of biofilm. When catheter replacement is not feasible, antifungal lock therapy, which gained considerable interest in the past decade, can be considered as an alternative option [122].

#### 7.2.8. Cleaning and decolonization of the patient

Currently, no specific intervention has been identified that can reduce or eliminate *C*. *auris* colonization [123]. Laboratory evidence indicates that high levels of chlorhexidine are active against *C*. *auris* [63]. In a study by Moore et al. [124], it was shown that a 70% isopropyl alcohol (IPA)-based skin disinfectant containing 2% chlorhexidine gluconate reduced *C*. *auris* counts to below detectable levels within 2 min (>5 log reduction). However, *C*. *auris* outbreaks and transmissions have been observed in healthcare facilities where chlorhexidine baths are routinely used. This underscores the significance of thorough infection control and prevention strategies^12^. Washing or wiping patients with chlorhexidine gluconate is an effective method to reduce both healthcare-associated infections and colonization with resistant organisms in the ICU [113,123]. Patients colonized or infected with *C*. *auris* are at risk for surgical site infections. Adherence to control measures is critical for preventing these infections. Unless contraindicated, skin preparation in the operating room should be performed using an alcohol-based agent [125].

### 7.3. Effectiveness of disinfectants on C. auris and disinfectant resistance

*C*. *auris* spreads through direct contact with colonized or infected patients, as well as through contaminated equipment and environmental contact [126]. Due biofilm formation, this fungus can remain on human skin, as well as on wet and dry environmental surfaces for weeks or even months [39,126]. Therefore, infection prevention and control protocols, along with cleaning and disinfection methods, play crucial roles in reducing the transmission of *C*. *auris* in healthcare settings [111,126].

Most disinfectants commonly used in hospitals that are effective against *C*. *albicans* and other fungi are not effective against *C*. *auris* because they cannot penetrate biofilms. In particular, disinfectants containing ammonia compounds solely are ineffective against this microorganism [111]. For the cleaning and disinfection of medical supplies and surfaces against *C*. *auris*, the CDC recommends using Environmental Protection Agency (EPA) approved products (List P) effective against *C*. *auris*. Thorough daily and terminal cleaning and disinfection of patient rooms and other areas where patients receive care (e.g., imaging, physical therapy, wound care, dialysis, etc.) should be conducted using these products^13^. Components of EPA-registered disinfectants effective against *C*. *auris* include:

- H_2_O_2_
- H_2_O_2_ and peracetic acid
- H_2_O_2_, peracetic acid, and octanoic acid
- Dodecylbenzenesulfonic acid
- Sodium hypochlorite
- IPA
- Quaternary ammonium compounds
- Sodium dichloro-s-triazinetrione
- Citric acid
- Ethanol, IPA, and didodecyldimethyl ammonium chloride

If access to CDC EPA-registered products effective against *C*. *auris* is not available, the CDC recommends the use of EPA-registered hospital-grade disinfectants that are effective against *Clostridium difficile* spores^14^. These disinfectants are available from various companies in the form of ready-to-use solutions, premade wipes, or dilutable preparations. It is important to adhere to the manufacturer’s usage instructions, including contact time, for all products. Additionally, touchless disinfection methods such as UV irradiation and vaporized H_2_O_2_ are recommended as complementary approaches to standard cleaning and disinfection methods^15^.

### 7.4. Management of waste and soiled linen

In hospitals, basic standard precautions should be followed while collecting, transporting, washing, storing, and reusing linens^15^, ^14^,^16^, ^17^, ^18^, ^19^.

#### 7.4.1. Management of soiled linens

- In the management of soiled linens, clean and dirty areas, as well as equipment, should be clearly defined. Personal protective equipment to be used in these areas and the infection control measures that must be followed should be specified [127].
- The collection of soiled laundry should be carried out in a manner that does not cause environmental contamination, minimizes aerosolization, and removes any foreign objects that could cause injury, ensuring that the partially clean side is facing outward [128].
- During the collection of soiled laundry, gowns and gloves should be worn. Hand hygiene must be practiced after any contact with soiled laundry [127,128].
- Soiled laundry should never be placed on the floor during the collection process and must be placed directly into the soiled laundry bag/cart without coming into contact with clean laundry [129].
- Sorting and counting of soiled laundry should not be performed in patient care areas during the collection process.
- If the laundry is wet and/or contaminated with blood or body fluids, it should be placed in leak-proof, water-soluble transparent bags, laundry bags, or puncture- and tear-resistant transport equipment chosen by the institution to prevent contact with the environment. The bags used for transferring the laundry should have labels indicating which unit they belong to [130].
- If soiled laundry is not to be taken immediately to the laundry washing area, it should be stored with a closed opening in designated soiled laundry holding areas and transferred to the laundry washing area in an appropriate manner as soon as possible.
- The laundry washing area should be organized to keep the acceptance of soiled textiles and the distribution of clean textiles separate from each other.
- The counted soiled laundry should be taken to the sorting point, where staff wearing waterproof gowns, masks, caps, shoes, gloves, and goggles (when necessary) should sort the laundry according to color and type [131,132].
- The antimicrobial activity of the washing process consists of a combination of mechanical, thermal, and chemical factors. The treatment of laundry with water removes a significant number of microorganisms. Hot water is an effective way to kill microorganisms [133].
- Laundry should be washed at a minimum of 71 °C for at least 25 min, and the temperature cycle should be recorded [134].
- If low-temperature (22 °C to 25 °C) laundry washing programs are used, chemicals suitable for low-temperature washing should be selected at the appropriate usage concentration [135].

#### 7.4.2. Management of medical wastes

Waste management and disposal should adhere to the CDC’s guidelines for environmental infection control in healthcare facilities and the regulations on the management of medical waste prepared by the Turkish Ministry of Environment, Urbanization, and Climate Change^20^,^21^.

Medical waste must be collected separately by personnel responsible for handling medical waste, apart from household, packaging, and hazardous waste. During the collection process, care should be taken to ensure medical waste bags and containers are labeled, and they must be delivered directly from the units. The medical waste collected from the units should be properly placed in temporary waste storage areas, and measures should be taken to prevent the accumulation of medical waste inside the units. Personnel responsible for collecting and transporting waste must wear appropriate clothing and personal protective equipment (PPE) for their work area and must ensure hand hygiene according to hand hygiene and glove use procedures.

Sharps and puncture-prone waste should be collected separately from other medical waste in puncture-, tear-, break-, and explosion-resistant, waterproof, and leak-proof plastic containers that cannot be opened or mixed. These containers must bear the international biohazard symbol and the label “CAUTION! SHARP AND CUTTING MEDICAL WASTE.” These collection containers should be filled to a maximum of ¾ full, securely closed, and placed in red plastic bags. Once the sharps containers are full, they must not be compacted, opened, emptied, or recycled. Liquid waste should be concentrated with appropriate absorbent materials and placed in waste bags. Waste transport vehicles used for transporting medical waste should be regularly cleaned and disinfected with steam disinfection after use. The storage area should be cleaned and disinfected after the waste has been emptied and treated with medication if necessary. In the event that a bag containing medical waste is torn or spills, the spilled waste should be collected with suitable equipment, and liquid waste should be concentrated with appropriate absorbent material before being placed back into a medical waste bag. The equipment used should be immediately disinfected in the storage area^22^, ^23^.

In the management of waste generated within the hospital and in the collection, transportation, preparation for use, storage, and distribution of soiled laundry and all types of textile products, the infection control instructions prepared within the hospitals must be followed.

### 7.5. Room cleaning upon patient discharge

The ability of *C*. *auris* to persist on surfaces such as fabrics, bedding, and curtains, along with its drug resistance and biofilm formation, increases the risk of transmission for patients and healthcare workers and makes its eradication from the hospital more difficult. There is a clear lack of information and methods in the guidelines published by global health organizations regarding decontamination. Currently, there is no single disinfectant that has been proven effective for all surfaces and materials. Therefore, research should continue not only on evidence-based decontamination methods but also on newer disinfectants.

In addition to manual cleaning and terminal disinfection, touchless disinfection technologies such as ultraviolet-C (UV-C), vaporized H_2_O_2_, and ozone are highly effective in disinfecting rooms contaminated with *C*. *auris*^24^ [136,137]. The selection of appropriate technology varies depending on the specific needs of the facility, the availability of equipment, and the nature of the contamination. It is always necessary to adhere to manufacturer instructions and safety guidelines when using these technologies.

#### 7.5.1. Terminal disinfection

Terminal disinfection of *C*. *auris* involves the comprehensive cleaning and disinfection of the patient’s room or healthcare area after the patient is discharged, transferred, or deceased. This process aims to remove organic material and significantly reduce or eliminate the fungal burden. By doing so, the spread of *C*. *auris* in healthcare facilities is prevented. Ensuring that this type of comprehensive disinfection is performed correctly each time requires rigorous training and diligent adherence to protocols^24^, ^25^.

- Manual cleaning and disinfection: As a first step, all removable items must be taken out of the room. Subsequently, terminal cleaning and disinfection should begin with shared equipment and common surfaces, followed by surfaces and items touched during patient care that are outside the patient area, and finally, surfaces and items that are directly touched by the patient within the patient area. In other words, high-touch surfaces outside the patient area should be cleaned before high-touch surfaces within the patient area^24^,^25^.
- Terminal disinfectants: It is recommended to use a product specifically approved by the EPA to disinfect surfaces contaminated with *C*. *auris*. A list of these products is provided in the relevant section.
- Terminal disinfection process^24^,^25^.

##### Precleaning

Visible dirt or organic material must be removed from surfaces and disposed of. Disposable cloths and mop heads should be used, or reusable items must be thoroughly cleaned after use.

##### Disinfection

An EPA-approved hospital-grade disinfectant effective against *C*. *auris* should be applied. The manufacturer’s instructions for concentration, contact time, and application method must be followed.

##### Focus areas

Special attention should be given to frequently touched surfaces, such as bed rails, overbed tables, nightstands, doorknobs, light switches, bathroom fixtures, and medical equipment.

##### Personal protective equipment

Cleaning staff must wear appropriate PPE, including gloves and gowns.

##### Waste management

Cleaning materials and all waste generated during the process must be disposed of properly.

#### 7.5.2. Use of vaporized H_2_O_2_

H_2_O_2_ provides a biocidal effect by harming crucial cellular elements like lipids, proteins, and DNA through the generation of free hydroxyl radicals. This agent displays broad-spectrum efficacy against numerous bacteria, fungi, and viruses. Nevertheless, the evidence supporting the effectiveness of H_2_O_2_ against *C*. *auris* is still considered moderate [123,138,139].

Experimental studies have shown that H_2_O_2_ is effective against *C*. *auris*, even at concentrations well below those used in hospital disinfectants [64,123,138,139]. Cadnum et al. [138] found that 0.5% and 1.4% H_2_O_2_ solutions were effective and noted that their lethality was comparable to that of chlorine-containing disinfectants. Interestingly, the authors mentioned that the 1.4% H_2_O_2_ disinfectant was effective with a contact time of 1 min. This is shorter than the 3 min recommended by the manufacturer. However, it was observed that the 0.5% concentration of H_2_O_2_ disinfectant still required the recommended contact time of 10 min for effective killing. H_2_O_2_ has been shown to be as effective against *C*. *auris* as it is against other *Candida* species, including *C*. *albicans*. Furthermore, no development of resistance to disinfection was observed even when *C*. *auris* colonies were repeatedly exposed to H_2_O_2_ over a span of 30 days [138,139]. Biswal et al. [64] reported effective environmental disinfection using H_2_O_2_ buffered with silver nitrate, but a contact time of 60 min was required for complete eradication, as recommended by the manufacturer.

In vitro studies have evaluated vaporized hydrogen peroxide (8 g peroxide/m^3^) on dried *C*. *auris* cells within a 96-well microtiter plate and found that it killed 96.6% to 100% of the *C*. *auris* isolates. It has been proposed that employing H_2_O_2_ vapor could be useful for decontaminating more complex healthcare devices, including ECG monitoring cables and blood pressure monitors and cuffs [126,140]. Furthermore, H_2_O_2_ vapor was used in conjunction with a chlorine-based cleaner (1000 ppm) as part of a routine disinfection protocol, and its effectiveness in managing *C*. *auris* was noted during an outbreak at a hospital in the United Kingdom [75].

#### 7.5.3. Ultraviolet application

An alternative method for decontaminating hospital surfaces is the use of mobile UV-C devices. The UK Public Health authority has recommended UV-C as a potential adjunct in managing the spread of *C*. *auris*^26^. However, evidence supporting its effectiveness is somewhat limited [141]. Cadnum et al. [142] showed experimentally that exposure to UV-C for 10 or 30 min reduced CFU from 40 to 1 × 10^6^, highlighting the significance of exposure time. In this investigation, samples were positioned 1.5 m away from the UV-C device. Since most hospital rooms exceed a 1.5 m radius, it is necessary to reposition the UV-C device after the initial cycle to ensure comprehensive coverage of the entire area. Further research examining distance and exposure time indicated that optimal UV-C effectiveness was achieved with 30 min of exposure at a maximum distance of 2 m from the UV-C device, suggesting that UV-C can effectively eliminate *C*. *auris*. However, several factors can influence the efficacy of UV-C application, including the density of *C*. *auris*, the duration of exposure to UV-C, and the distance to the UV source [141]. In another study, where samples were situated 1 m from the UV-C light and directly facing the lamp, all *C*. *auris* were eradicated after 15 min of exposure [143]. Additionally, a recent investigation into the effectiveness of 254 nm UV-C light in inactivating *C*. *auris* on hard surfaces utilized a mobile UV-C tower equipped with high-performance bulbs. In a patient-room-sized test environment, a continuous exposure of 7 min resulted in ≥99.97% (≥3.86 log_10_) inactivation of *C*. *auris* [137]. These results suggest that UV-C could act as an adjunct infection control strategy against *C*. *auris* in healthcare environments.

It is also recognized that UV-C can prevent the formation of *C*. *auris* biofilms. This has been studied using stainless steel, plastic coupons, and polycotton fabric. The highest susceptibility to UV-C disinfection was found within the wavelength range of 267 to 270 nm. A dose of 30 mJ/cm^2^ effectively inhibited biofilm formation on stainless steel coupons, whereas 10 mJ/cm^2^ was necessary for plastic surfaces. A 60 mJ/cm^2^ dose significantly diminished biofilms on polycotton fabric [144].

Although incorporating H_2_O_2_ vapor or UV-C light to enhance disinfection during terminal cleaning may offer additional benefits [123,126], further data on their applications is required.

## 8. SURVEILLANCE RECOMMENDATIONS FOR *C*. *auris*

### 8.1. Emergence of the need for surveillance

Between 2013 and 2017, sporadic cases of *C*. *auris* were reported in the European Union and the European Economic Area (EU/EEA). From 2018 onwards, a significant increase in the number of cases and outbreaks has been observed^27^ [7]. Therefore, the necessity for ensuring adequate laboratory capacity for the early detection of *C*. *auris* and the rapid implementation of control measures has become evident [15].

In 2018, the ECDC conducted a survey concerning laboratory capacity and preparedness related to *C*. *auris*, which was sent to EU/EEA countries. Among the 29 countries that responded, 21 had the ability to detect and diagnose *C*. *auris*. Twelve of these countries had officially assigned mycology reference laboratories, while nine had laboratories functioning as reference centers. Public health measures for preparedness or response to *C*. *auris* were reported in 20 countries. The most frequently mentioned measures included the dissemination of laboratory alerts (18 countries), dissemination of clinical alerts (10 countries), and notifications regarding reference identification and antifungal susceptibility testing in hospital labs (13 countries). It was also observed that the preparation of guidelines for laboratory testing (7 countries), clinical management (4 countries), and infection control (4 countries) occurred less often, and retrospective or prospective surveillance was available in only a limited number of countries (8 and 7 countries, respectively)^28^.

### 8.2. Emergence of the need for surveillance in Türkiye

Since the initial reports from the EU/EEA, the Turkish National Reference Laboratory has raised awareness among healthcare personnel regarding the identification of *C*. *auris*. Suspected *C*. *auris* isolates began to be sent to the NMRL-PHIT from the ICUs of various hospitals for identification and antifungal susceptibility testing for the first time during the COVID-19 pandemic in June 2020. Since then, there has been an increase in the number of hospitalized patients with *C*. *auris* colonization or invasive infection, reaching a total of 13 hospitals that have reported such cases.

The hospitals referring their cases to the NMRL-PHIT on a voluntary basis conduct colonization screening, establish hospital-specific antifungal susceptibility profiles, and monitor resistance. In outbreak situations, colonization screening may also be performed on commonly used devices and hospital environments.

### 8.3. Current laboratory infrastructure

The WHO recommends developing mycology diagnostic capacity starting from reference microbiology laboratories for the identification of fungi and susceptibility testing, in order to manage fungal infections and conduct surveillance^27^. Since 2015, diagnostic and antifungal susceptibility testing conducted at the NMRL-PHIT as well using reference methods according to ISO 15189 standards, and molecular diagnostic capacity has also been improved. Additionally, with contributions from the WHO and the EU, a project conducted at PHIT has resulted in the construction of the Türkiye National Genomic Surveillance Strategy 2023–2028. Among the pathogens on this priority list, *C*. *auris* was selected as the top concern from a mycological perspective for Türkiye. This has led to the establishment of laboratory infrastructure for this pathogen and the initiation of studies aimed at identification and resistance monitoring through whole-genome sequencing analysis.

### 8.4. Recommendations for developing surveillance infrastructure in Türkiye

#### 8.4.1. Antifungal susceptibility testing

Currently, the recommended reference methods for determining antifungal susceptibility profiles and identifying the MIC values for fungal agents are microdilution-based methods, as outlined by CLSI and EUCAST. However, few microbiology diagnostic laboratories in Türkiye are able to implement reference microdilution methods for antifungal susceptibility testing. The number of experts and staff working in mycology is limited in the country, similar to the situation worldwide. Therefore, it would be appropriate to conduct surveys that target identification of fungal infections, antifungal susceptibility analysis, and outbreak detection to assess the capacity of microbiology laboratories in the country.

#### 8.4.2. Laboratory capacity targets

To enhance the diagnostic capacity for *C*. *auris* infections, increasing the mycological diagnostic capacity in other microbiology laboratories in Türkiye should be a priority public health goal.

#### 8.4.3. National *C. auris* surveillance network

The decision to report patients infected or colonized with *C*. *auris* to the National Health Service-Associated Infections Surveillance Network has been announced by the Ministry of Health of the Republic of Türkiye with a letter dated September 10, 2024, and numbered E-27249879-771-253094204. Isolates are requested to be sent to the NMRL-PHIT for confirmation of microbiological diagnosis, molecular detection of antifungal resistance, and clade determination.

## 9. HANDS-ON TRAINING

### 9.1. Planning hands-on training

The hands-on training on *C*. *auris* should target a diverse audience, including infectious disease fellows, specialists, physicians from various departments, intensive care nurses, infection control nurses, and cleaning personnel. The training content should be customized to the participants’ levels. When forming smaller discussion or practical groups, consider the diversity of the participants.

To align the training framework with the SMART criteria, the planning process should address the following elements^29^ [145,146]:

- **Specific**: Clearly define the educational objectives participants will achieve by the end of the training
- **Measurable**: Establish measurable outcomes to assess participant learning at the conclusion of the training
- **Attainable**: Develop a training plan that allows participants to meet objectives within the planned timeframe without feeling rushed
- **Relevant**: Ensure the training addresses the needs of participants and the organization
- **Timely**: Clearly outline the timeline for achieving objectives

### 9.2. Selection of educators

Educators should possess expertise and firsthand experience in the challenges associated with *C*. *auris* and related practices. It is essential to involve clinicians with specific case experience and, if applicable, laboratory specialists, academics, or researchers familiar with *C*. *auris* and relevant molecular methods. The training team may include experts in infectious diseases, clinical microbiology, and infection control.

Educators should convene at least once before the training to ensure their lessons are coordinated and complementary^30^ [145,146].

### 9.3. Development of training materials

Training materials should be prepared based on the target participant group, including:

- Lecture notes and presentations
- Articles
- Case studies
- Theoretical and practical assessment questions
- Laboratory materials
- Brochures

Well-prepared materials are crucial for effective training. Key points should be emphasized during presentations, while supplementary content should be provided for participants to review independently, enhancing the effectiveness of the training.

### 9.4. Recommended topics for hands-on training on *C. auris*

Prioritized topics for the training should include^30^, ^31^, ^32^ the following.

#### 9.4.1. General Information about *C. auris*

- Routes of transmission
- Infections caused by *C*. *auris*
- Importance of awareness

#### 9.4.2. Infection control and prevention

- Infection control guidelines
- Environmental control measures
- Disinfection procedures
- Management of *C*. *auris* in dialysis units and outpatient centers
- Infection control in ICUs
- Patient transfer considerations

#### 9.4.3. Diagnosis and Treatment

- Diagnosis and susceptibility testing
- Criteria for treatment initiation
- General treatment approaches
- Management of resistant *C*. *auris*

#### 9.4.4. Case Discussions, simulations, and small group workshops

- Case examples from intensive care patients
- Cases involving comorbidities
- Challenging resistant cases

#### 9.4.5. Surveillance

### 9.5. Primary objective of the training

The delivery of the curriculum is as important as its content. Interactive teaching methods and effective communication strategies tailored to participants are vital for success. By the end of the program, participants should be well-informed about *C*. *auris* and capable of recognizing necessary cleaning practices and infection control measures. To promote ongoing education, regular training sessions should be scheduled [146].

## Preface

Today, numerous microorganisms are triggering outbreaks, prompting us to reassess our knowledge and practices. While we deal with challenges posed by certain hospital-acquired bacteria that are hard to control and show resistance to multiple antibiotics, a new fungus, *Candida auris*, has rapidly emerged among problematic microorganisms. This organism is significant due to its potential to cause severe infections, resistance challenges, and rapid dissemination. Easy transmission through contact makes infection control using standard procedures difficult.

As the Infectious Diseases and Clinical Microbiology Specialty Society of Türkiye (EKMUD), we have decided to prepare a consensus report regarding this pathogen, which is also found in our country. The goal of this report is to standardize the knowledge of physicians nationwide regarding *C*. *auris*, outline important parameters for diagnosing and monitoring the disease, promote effective treatment options, and assess preventive measures. To achieve this, we have partnered with the nation’s health authorities and relevant societies. We completed this report in five months with contributions from the Ministry of Health of the Republic of Türkiye, the Clinical Microbiology Specialty Association (KLIMUD), the Turkish Microbiology Society (TMC), the Turkish Society of Anesthesiology and Reanimation (TARD), the Turkish Society of Intensive Care (TYBD), the Infectious Diseases International Research Initiative (ID-IRI), and all our collaborators. We extend our gratitude to our colleagues who supported this initiative despite their demanding schedules.

As you review the consensus report, you will find answers to many of your questions. It covers topics such as the epidemiology, microbiology, treatment options, screening strategies, infection control measures, surveillance, and educational aspects related to the infection and colonization of the microorganism. Since information about *C*. *auris* may evolve, our colleagues have agreed on future updates to the report.

We hope this serves as a valuable resource for all of us.

Prof. Canan AĞALAR, MD

Prof. Hakan ERDEM, MD

Prof. Yasemin ÇAĞ, MD

## Figures and Tables

**Figure:**
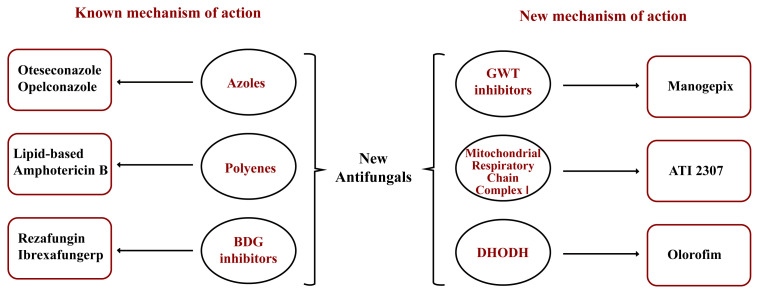
New antifungals and their mechanisms of action. BDG: Beta-D-glucan; GWT 1: Fungal acetyltransferase enzyme; DHODH: Dihydroorotate dehydrogenase.

**Table 1:** The MIC ranges of *C*. *auris* isolates sent to NMRL-PHIT between June 2020 and August 2023.

	MIC range *	MIC 50	MIC 90	Number of isolates
Amphotericin B	0.5–4	2	4	69
Fluconazole	64–256	256	256	69
Itraconazole	0.125–16	0.5	2	54
Voriconazole	0.125–8	0.25	1	69
Caspofungin	0.03–32	0.125	1	67
Anidulafungin	0.06–4	0.25	1	51

* Tested by CLSI microdilution method.

**Table 2:** Efficacy of new antifungals in fungal infections.

	Prophylaxis	Vaginitis	Candidemia	Aspergillosis	Cryptococcosis	Others
Opelconazole	++ Lung tx, cystic fibrosis	+	+	++	−	−
Oteseconazole	+	++	+	+	+	+
L-AmB	−	−	+	+	++	−
Rezafungin	++	−	++	+	−	+
Ibrexafungerp	++	++	++	++	−	+
Manogepix	+	+	++	++	+	++
Olorofim	−	−	−	++	−	++

L-AmB: Liposomal Amphotericin B; (−): No clinical studies exist, and considering the in vitro activity and pharmacokinetic characteristics, an indication seems improbable; (+): There are no clinical studies available. However, an indication may be possible based on in vitro activity and pharmacokinetic characteristics; (++): Clinical studies are available, and an indication is supported by in vitro activity, pharmacokinetic characteristics, and clinical data.

**Table 3:** Performance metrics in the operation of the AFS program.

Parameter	Description
Antifungal consumption	Regular measurements of DDD or DOT
Requests/prescribing	The rationale for treatment should be recorded Therapeutic drug levels (for voriconazole) should be monitored
Treatment deescalation	Review of the necessity for continuation of empirically initiated treatment Transition to a narrow spectrum based on susceptibility results and clinical response (e.g., fluconazole) Switching from intravenous treatment to oral administration in patients without bioavailability problems
Diagnostic tests	Timely testing for the necessary diagnostics in suspected invasive fungal disease (thus allowing for treatment modification or discontinuation if necessary)
Development of local guidelines or algorithms	Diagnosis, treatment, and monitoring algorithms should be developed according to the center’s conditions in light of widely accepted national and international guidelines. Adherence to these guidelines should be periodically audited, and feedback should be provided.

DDD: Defined daily dose; DOT: Days of therapy.

## Footnotes

1 World Helath Organization (2022). WHO fungal priority pathogens list to guide research, development and public health action [online]. Website https://iris.who.int/bitstream/handle/10665/363682/9789240060241-eng.pdf?sequence=1 [accessed 23 July 2024].

2 Karabıçak N, Tekçe Y, Kömeç S, Tekinaslan N, Bayraktar F et al. (2022). Increased cases of *Candida auris* infection from various hospitals in Turkey during the COVID-19 pandemic. In: 32nd ECCMID. Portugal

3 The MIC values of all C. auris isolates sent to the Public Health Institution of Türkiye, National Mycology Reference Laboratory between June 2020 to August 2023. (2023) (Unpublished data).

4 US Centers for Disease Control and Prevention (2024). *Candida auris* antifungal susceptibility testing [online]. Website https://www.cdc.gov/candida-auris/hcp/laboratories/antifungal-susceptibility-testing.html [accessed 2 August 2024].

5 WHO fungal priority pathogens list to guide research, development and public health action (2022). [online], Website https://iris.who.int/bitstream/handle/10665/363682/9789240060241-eng.pdf?sequence=1, [accessed 23 July 2024]

6 World Health Organization (2022).WHO fungal priority pathogens list to guide research, development and public health action [online]. Website https://iris.who.int/bitstream/handle/10665/363682/9789240060241-eng.pdf?sequence=1, [accessed 23 July 2024].

7 Sikora A, Hashmi MF, Zahra F. Candida auris (2023). StatPearls Publishing, Treasure Island (FL) [online]. Website https://www.ncbi.nlm.nih.gov/books/NBK563297/ [accessed 23 July 2024].

8 World Health Organization (2022). WHO fungal priority pathogens list to guide research, development and public health action [online]. Website https://iris.who.int/bitstream/handle/10665/363682/9789240060241-eng.pdf?sequence=1, [accessed 23 July 2024].

9 Sikora A, Hashmi MF, Zahra F (2023). Candida auris. StatPearls Publishing, Treasure Island (FL) [online]. Website https://www.ncbi.nlm.nih.gov/books/NBK563297/ [accessed 23 July 2024].

10 Infection Control Guidance: Candida auris (2024). [online], Website https://www.cdc.gov/candida-auris/hcp/infection-control/index.html. [accessed 1 July 2024]

11 European Centre for Disease Prevention and Control (2016). Candida auris in healthcare settings [online]. Website https://www.ecdc.europa.eu/sites/default/files/media/en/publications/Publications/Candida-in-healthcare settings_19-Dec-2016.pdf [accessed 23 July 2024].

12 U.S. Centers for Disease Control and Prevention (2024). Infection Control Guidance: *Candida auris* [online]. Website https://www.cdc.gov/candida-auris/hcp/infection-control/index.html [accessed 1 July 2024].

13 United States Environmental Protection Agency (2024). EPA’s Registered Antimicrobial Products Effective Against Candida auris [online]. Website https://www.epa.gov/pesticide-registration/epas-registered-antimicrobial-products-effective-against-candida-auris-list#against [accessed 23 July 2024].

14 United States Environmental Protection Agency (2024). EPA’s Registered Antimicrobial Products Effective Against Clostridioides difficile Spores [online]. Website https://www.epa.gov/pesticide-registration/epas-registered-antimicrobial-products-effective-against clostridioides [accessed 23 July 2024].

15 U.S. Centers for Disease Control and Prevention (2024). Infection Control Guidance: *Candida auris* [online]. Website https://www.cdc.gov/candida-auris/hcp/infection-control/index.html [accessed 1 July 2024].

16 European Centre for Disease Prevention and Control (2016). Candida auris in healthcare settings – ECDC [online]. Website https://www.ecdc.europa.eu/sites/default/files/media/en/publications/Publications/Candida-in-healthcare settings_19-Dec-2016.pdf [accessed 23 July 2024].

17 U.S. Centers for Disease Control and Protection (2024). Laundry and Bedding [online]. Website https://www.cdc.gov/infection-control/hcp/environmental-control/laundry-bedding.html [accessed 1 August 2024].

18 UK Health Security Agency (2023). Candida auris: laboratory investigation, management and infection prevention and control (draft) [online]. Website https://www.gov.uk/government/consultations/candida-auris-update-to-management-guidance/candida-auris-laboratory-investigation-management-and-infection-prevention-and-control-draft [accessed 10 November 2024].

19 US. Centers for Disease Control and Prevention (2024). Linen and laundry management [online]. Website https://www.cdc.gov/healthcare-associated-infections/hcp/cleaning-global/appendix-d.html [accessed 1 August 2024].

20 Tıbbi Atıkların Kontrolü Yönetmeliği Resmi Gazete Sayısı (2017), [online]. Website https://www.resmigazete.gov.tr/eskiler/2017/01/20170125-2.htm [accessed 10 November 2024]

21 U.S. Centers for Disease Control and Prevention (2024). Regulated Medical Waste [online]. Website https://www.cdc.gov/infection-control/hcp/environmental-control/regulated-medical-waste.html [accessed 1 August 2024].

22 Tıbbi Atıkların Kontrolü Yönetmeliği Resmi Gazete Sayısı (2017), [online]. Website https://www.resmigazete.gov.tr/eskiler/2017/01/20170125-2.htm [accessed 10 November 2024]

23 U.S. Centers for Disease Control and Prevention (2024). Regulated Medical Waste [online]. Website https://www.cdc.gov/infection-control/hcp/environmental-control/regulated-medical-waste.html [accessed 1 August 2024].

24 U.S. Centers for Disease Control and Prevention (2024). Environmental Cleaning Procedures [online]. Website https://www.cdc.gov/healthcare-associated-infections/hcp/cleaning-global/procedures.html [accessed 1 July 2024].

25 6 U.S. Centers for Disease Control and Prevention (2024). Infection Control Guidance: Candida auris [online]. Website https://www.cdc.gov/candida-auris/hcp/infection-control/index.html [accessed 1 July 2024].

26 UK Health Security Agency (2017). Guidance for the laboratory investigation, management and infection prevention and control for cases of Candida auris [online]. Website https://assets.publishing.service.gov.uk/media/5a822c35e5274a2e87dc1869/Updated_Candida_auris_Guidance_v2.pdf [accessed 10 November 2024]

27 World Health Organization (2022). WHO fungal priority pathogens list to guide research, development and public health action [online]. Website https://iris.who.int/bitstream/handle/10665/363682/9789240060241-eng.pdf?sequence=1 [accessed 23 July 2024].

28 European Centre for Disease Prevention and Control (2016). Candida auris in healthcare settings [online]. Website https://www.ecdc.europa.eu/sites/default/files/media/en/publications/Publications/Candida-in-healthcare-settings_19-Dec-2016.pdf [accessed 23 July 2024]

29 World Health Organization (2022). WHO fungal priority pathogens list to guide research, development and public health action [online]. Website https://iris.who.int/bitstream/handle/10665/363682/9789240060241-eng.pdf?sequence=1 [accessed 23 July 2024].

30 Evenium (2018). Ways to Create Captivating Physician Conferences [online]. Website https://corp.evenium.com/10-ways-to-create-captivating-physician-conferences/ [accessed 6 July 2024].

31 U.S. Centers for Disease Control and Prevention (2024). Infection Control Guidance: Candida auris [online]. Website https://www.cdc.gov/candida-auris/hcp/infection-control/index.html [accessed 1 July 2024].

32 Fungus Education Hub (2024). Mycoses Study Group, Education & Research Consortium [online]. Website https://funguseducationhub.org/candida-auris-prevention-and-management-a-case-based-course/ [accessed 5 July 2024].

## List of Abbreviations

EU: European Union
EEA: European Economic Area
VHP: Vaporized hydrogen peroxide
CDC: Centers for Disease Control and Prevention
CLSI: The Clinical and Laboratory Standards Institute
WHO: World Health Organization
ECDC: European Centre for Disease Prevention and Control
EPA: Environmental Protection Agency
EUCAST: The European Committee on Antimicrobial Susceptibility Testing
FDA: U.S. Food and Drug Administration
ITS: Internal transcribed spacer
IPA: Isopropyl alcohol
ISO: International Organization for Standardization
CRB: Clinical resistance breakpoints
PPE: Personal protective equipment
MALDI-TOF MS: matrix assisted laser desorption ionization time of flight mass spectrometry
MIC: Minimum inhibitory concentration
PCR: Polymerase chain reaction
NMRL: National Mycology Reference Laboratory
PHIT: Public Health Institution of Türkiye
ICU: Intensive care unit
